# Localization and dynamic change of saponins in *Cyclocarya paliurus* (Batal.) Iljinskaja

**DOI:** 10.1371/journal.pone.0223421

**Published:** 2019-10-15

**Authors:** Xiaoling Chen, Yu Wang, Hu Zhao, Xiangxiang Fu, Shengzuo Fang

**Affiliations:** College of Forestry, Nanjing Forestry University Southern Modern Forestry Collaborative Innovation Centre, Nanjing Forestry University, Nanjing, China; National University of Kaohsiung, TAIWAN

## Abstract

*Cyclocarya paliurus* is a unique tree species of that grows in southern China. The tree contains distinctive saponins in the leaf that has hypoglycemic and hypolipidemic effects. It was aimed to detect localization of saponins and suitable time of harvest for medicinal uses. Histochemical, cytochemical localization and UV-spectrophotometry were carried out in *C*. *paliurus* plant. We found that in all organs, the saponins were primarily located in the parenchyma cells and the highest saponins accumulation was in the palisade tissue in leaves. Cytochemical localization results indicated that saponins were mainly distributed in the chloroplast, vesicle, and plasmalemma. On average, the total saponins content in leaves (20.57 mg·g^-1^) was two and three times greater than in root (10.19 mg·g^-1^) and shoot (6.20 mg·g^-1^), respectively. Moreover, the saponins content in the leaf and root exhibited fluctuations, which were highest in September. Considering saponins levels and biomass, we conclude that harvesting all leaves in September is an economical and effective strategy for medicinal use in *C*. *paliurus*.

## Introduction

*Cyclocarya paliurus* is widely distributed in the southern region of China and is commonly known as Qing-Qian-Liu for its fruit clusters that look like a string of old Chinese copper coins [1]. Previous studies have found that *C*. *paliurus* contains a variety of bioactive substances, among which are saponins. Saponins are known to have a wide range of pharmacological properties, including anti-inflammatory, anti-tumor, anti-hyperglycemic, and anti-hyperlipidemic effects [2–5]. Saponins have also been found to boost the immune system and reduce high blood pressure [2,6]. There are several distinctive saponins that have been isolated and identified from *C*. *paliurus* leaves, including cyclocarioside A and cyclocarioside I, which are 200 and 250 times as sweet as sucrose, respectively [7,8]. To date, most studies involving saponins from *C*. *paliurus* have mainly focused on the pharmacological function of saponins on diabetes [9–11] and hyperlipidemia [12]. Studies have also investigated the separation and purification of active compounds [13] and the extraction and identification of new compounds [14]. However, little is known about the storage and localization of saponins in the *C*. *paliurus* plant.

In recent years, histochemical localization has been used to fix accumulating position of secondary metabolites in Chinese medicinal materials. According to types of composition, different methods have been used to determine cellular localization of alkaloids [15], saccharides [16], saponins [17], quinones, and flavonoids [16,18]. Research investigating the locations of saponins in *Panax ginseng*, *Bupleurum chinense*, and *Polygala tenuifolia* by vanillin-HAc and perchloric acid staining has found that saponins accumulated primarily in the parenchyma cells in the secondary phloem, cortex, and periderm of the root and shoot, as well as the epidermal and mesophyll cells in leaves [17,19,20]. However, differences in saponins accumulation among organs indicated that the distribution and types of saponins largely depend on species and tissues [21]. The initial saponins extraction from leaves is typically conducted by adding ammonium sulfate, lead acetate or another neutral salt to an aqueous solution of acid saponins [22]. Then Cai et al. (2009) proposed an approach that used lead acetate precipitation to localize the saikosaponin of *Bupleurum scorzonerifolium* at the subcellular level, through which, they found that saikosaponin mainly located in cytoplasm of epidermal cells, especially in vacuole, in addition, saponins were also found in the plasmodesma and golgi apparatus [23].

As a medicinal woody plant, harvest season and harvest organ are vital for *C*. *paliurus*. However, which organ (leaf, stem or root) is the main location for saponins synthetic? And what season is the optimum for saponins accumulation? To determine the suitable harvest strategy, histochemical and cytochemical analyses were used to detect saponins content in various vegetative organs across growing seasons. Combined with previous work on the biomass of *C*. *paliurus*, we aim to provide supports to determine harvest strategy for medicinal use of *C*. *paliurus*. Meanwhile, the study also can make foundation to understand the pathways of synthesis and transport of saponins; more importantly, it will provide a basis for developing a strategy of cultivation for medicinal use.

## Materials and methods

### Plant materials

No specific permissions were required for the location. The field studies did not involve endangered or protected species.

Samples for histochemical and saponins content determinations were collected from 6-year-old *C*. *paliurus* trees that had previously been established as a trial field in Zhenjiang, Jiangsu Province, China (119°32'E, 32°16'N). Current year shoot, leaflets, and mature lateral roots were collected at the end of April, May, June, July, and September of 2016. The four individuals in every replicates selected in this study were average sample trees determined by tree height and breast diameter, and there were three replications. Root samples were rinsed with running deionized water.

From a separate and second source of *C*. *paliurus*, samples were collected for cytochemical localization. These samples were collected from yearling that had been cultivated in a greenhouse (25 ± 2°C, relative humidity at 60–70%) in Nanjing Forestry University, Nanjing, Jiangsu Province, China (118°48'E,32°04'N). Tender leaves were collected in April, and fully developed were collected in May. Sample came from one individual was regarded as one replicate, and there were three replications.

### Histochemical localization

Histochemical localization was carried out according to methods outlined by Teng et al. (2009) and Tan et al. (2008) [17,20]. Sections of fresh tissue 25–35 μm in length were cut using a freezing microtome Leica-CM1850 (Leica, Beijing, China), treated with 5% lead acetate for 10 min to precipitate saponins from the tissue, and then stained with a mixed solution of 5% vanillin-HAc and perchloric acid (v:v = 1:1) for 5–10 min. Treated sections were then observed and photographed under a light microscope Olympus CX-41 (Olympus, Tokyo, Japan). The control samples were fixed in formalin-aceticacid-70% alcohol (FAA) for 30 days to remove saponins. Sections were treated and stained as frozen sections. Ten sections were randomly selected from each sample for observation.

### Cytochemical localization

Leaflets were treated following published protocols [23]. Leaflet samples were cut into 1 mm^3^ fragments and fixed with 2% glutaraldehyde (prepared with 3% lead acetate in 0.1 M sodium cacodylate buffer at a pH of 7.2) for 4 h at 4°C. Samples were then washed four times for 30 min each time with 3% lead acetate, following which, the cuts were incubated with 1% osmic acid (prepared with 3% lead acetate in 0.1 M sodium cacodylate buffer at a pH of 7.2) overnight at 4°C, then washed twice (each for 30 min) with 0.1 M sodium cacodylate buffer at a pH of 7.2. The fragments were then gradually dehydrated with a grade series of ethanol of 30%, 50%, 75%, 85%, 95%, and 100%. Each ethanol step was exposed to the sample for 30 min. Next, samples were incubated with epoxy propane transition and embedded with Epon812. The fragments of the leaflet were cut into sections 50–70 nm long using an ultramicrotome LKB-V (LKB, Sweden). After samples were stained with uranyl acetate, the sections were subsequently observed and photographed using a transmission electron microscope HITACHI 7650 (HITACHI, Tokyo, Japan). The control sample was treated with the same procedures as described above, but the fixing step with lead acetate was excluded. Ten sections were randomly taken from each sample for observation.

### Determination of total saponins

A total of 0.500 g dried powder of tissue (leaflets, shoots, roots) of *C*. *paliurus* was degreased with petroleum ether twice at 80°C, 4 h for each time [24]. The defatted retained residue was air-dried at ambient temperature. Total saponins in the residue were extracted using an ultrasonic-assisted method [25]. Briefly, the residue was soaked with 10 ml ethanol solvent (80%, v/v) for 12 h, and sonicated (KQ250B, Kunshan Ultrasonic Instruments Co., Ltd., Kunshan, China) at 59 kHz for 40 min at 70°C, then filtered through 0.45 μm microporous membrane. The above process was repeated. Two extracts were combined, condensed under low pressure, and adjusted to 10 ml with 100% ethanol.

The total saponins content was determined by using UV spectrophotometry UV-4802H (Unico, American) at a wavelength of 550 nm in accordance with the protocol described by Deng et al. [26]. A 5% vanillin-glacial acetic acid-perchloric acid solution stained reagents, calculated by the standard oleanolic acid (Nanjing Zelang Medical Technology Co. Ltd., Nanjing, China) curve, expressed as milligrams of oleanolic acid equivalent per gram of dry weight (mg·g-1).

### Statistical analysis

Data were presented as mean ± standard deviation. One-way analysis of variance (ANOVA) were conducted to test the significance of total saponins accumulation by Bonferroni test. All statistical analyses were performed at a 95% confidence level. Calculations were conducted using SPSS 20.0 software (SPSS Inc., Chicago, IL, USA).

## Results

### Histochemical localization of saponins in vegetative organs

**Fig 1:**
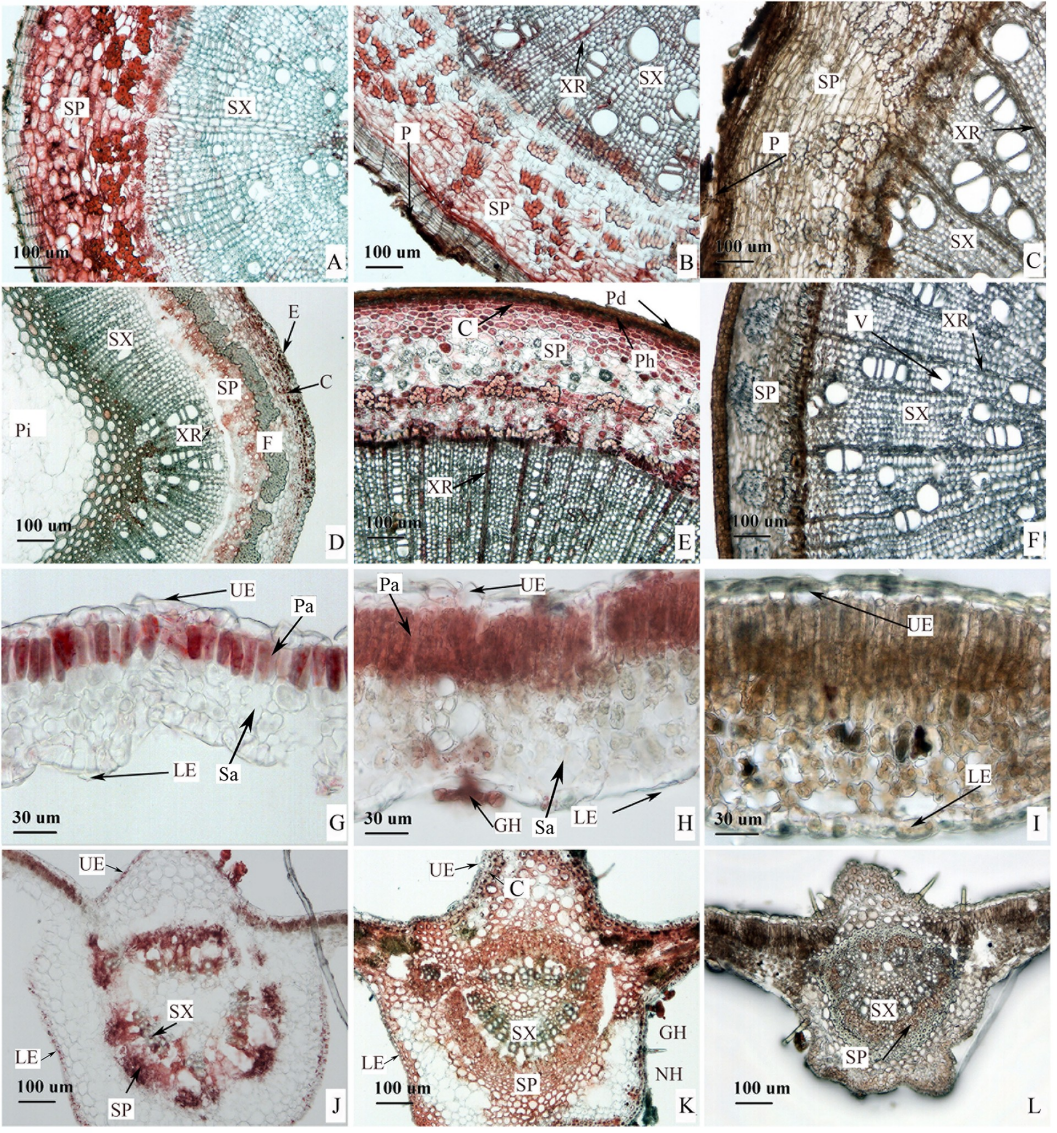
Histochemical localization of saponins in *C*. *paliurus*. (**A**) Cross section of new roots collected in April. Secondary phloem is displayed by deep red; (**B**) Cross section of mature roots collected in May. Phelloderm is indicated with a deep red, and secondary phloem and xylem ray are light red; (**D**) Cross section of shoots collected in May. Epidermis and cortex are indicated with deep red, and the phloem and xylem ray in light red; (**E**) Shoot collected in September. Phelloderm of periderm is in red; (**G**) Cross section of leaf (April). Mesophyll tissue is displayed by red, and epidermis is displayed by light red; (**H**) Cross section of leaf (June). Palisade parenchyma and glandular hair are deep red, while epidermis is light red; (**J**) Cross section of the midrib in tender leaf (May). Undifferentiated phloem is indicated with red; (**K**) Cross section of the midrib in mature leaf. Upper epidermis and cortex are indicated with deep red (May); (**C**, **F**, **I**, **L**) The control sample of root, shoot, leaf and midrib, respectively. GH, Glandular hair; LE, Lower epidermis; NH, Nonglandular hair; SP, Secondary phloem; SX, Secondary xylem; UE, Upper epidermis; C, Cortex; E, Epidermis; F, Fiber; P, Periderm; Pd, Phelloderm; Ph, Phellem; Pi, Pith; XR, Xylem ray; Pa, Palisade tissue; Sa, Spongy parenchyma.

Saponins can exhibit characteristic colors from light red to dark red when they react with a 5% vanillin-glacial acetic acid-perchloric acid solution [27]. Thus, the distribution and the accumulation of saponins can be judged according to the change of color. In our study, the distribution patterns of saponins in mature lateral roots were quite different between new and old roots (Fig 1A and 1B). In the new root, secondary phloem contained massive saponins (dark red). In the phelloderm of the periderm, parenchyma cells in the central and vascular cambium were light red, indicating small quantities of saponins. No color appeared in phellem layer and secondary xylem (Fig 1A), suggesting there were no saponins present in these locations. In the old roots, saponins found in phelloderm were concentrated, and those found in the secondary phloem were scattered. Meanwhile, red also occurred in the xylem ray located in the secondary xylem (Fig 1B). The distribution pattern in other parts was similar to that in the new root (Fig 1A).

In young shoot (April), dark red continuous bands were detected in the epidermis, and dispersal particles were found in the cortex parenchyma. Pale red was identified in the secondary phloem and xylem ray, and no color was evident in other parts of tender cuttings (Fig 1D). In the mature shoot (September), a purple-red color was primarily distributed in phelloderm, cortex, and secondary phloem. Light red was evident in the xylem ray, and no color emerged in other section of the shoot (Fig 1E).

In leaves, deep red primarily occurred in palisade tissue of both the tender and mature leaflets, indicating that a significant quantity of saponins accumulated in these locations (Fig 1G and 1H). The thickness and density of the palisade tissue in the mature leaflets were greater than tender leaves (Fig 1G), predicating that more saponins are accumulated in mature leaflets. However, few saponins were found in the spongy tissue of both leaves. Saponins were also found in the primary vein in the mature leaflet. The secondary phloem, epidermis, and cortex were deep red, and the secondary xylem of the mature leaflet was pale red (Fig 1K). Dark red only occurred in undifferentiated phloem of the main vein in tender leaflets, and no color was found in other parts (Fig 1J). Controls of root, shoot, leaf, and main vein (Fig 1C, 1F, 1I and 1L) did not produce any characteristic red when reacting with the chromogenic agent.

### Cytochemical localization of saponins in leaves

**Fig 2:**
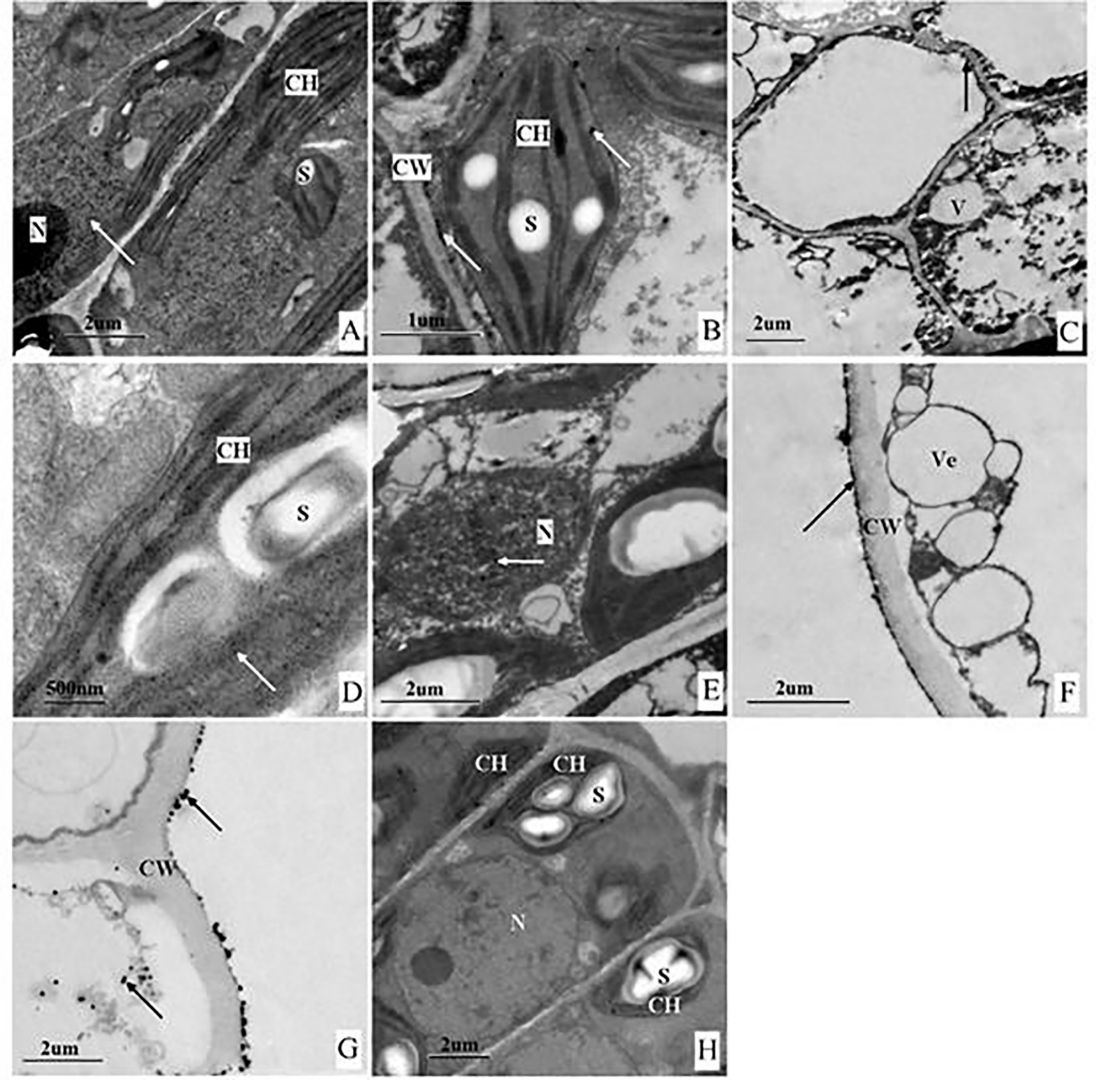
Cytochemical localization of saponins in leaves of *C*. *paliurus*. Saponin precipitations is black masses indicated with an arrow. (**A**) Nucleus (young leaf); (**B**) Chloroplast (young leaf); (**C**) Epidermal cell (young leaf); (**D**) Chloroplast (mature leaf); (**E**) Nucleus (mature leaf); (**F**) Vesicle and plasmalemma (mature leaf); (**G**) Epidermis cell wall and the cytoplasm (mature leaf); (**H**) Control with no saponins precipitations. CH, Chloroplast; CW, Cell wall; N, Nucleus; S, Starch grains; V, Vacuole; and Ve, Vesicle.

In mesophyll cells of tender leaflets, the quantity of black granules (a proxy for saponins) [23,28] was observed in cytoplasm, and the undeveloped chloroplast also contained a limited number of saponins (Fig 2A and 2B). However, in mature mesophyll cells, saponins mainly accumulated in the chloroplast alongside starch granules (Fig 2D). In the epidermal cells of both the tender and mature leaflets, saponins were located in the cytoplasm adjacent to the cell wall (Fig 2C and 2F). However, no saponins were found in the cell wall. Saponins on the endoplasmic reticulum vesicles were found in mature leaflets (Fig 2F), and many black granules occurred outside the cell wall (Fig 2G). Black granules also occurred in the nucleus (Fig 2A and 2E). When leaves matured from April to September, the quantity of black granules and starch grains increased in the chloroplast and decreased in the nucleus.

### Dynamics of total saponins content in organs during the growing months

Lateral roots, shoots, and leaflets were taken at the end of each month from April to September. The content of total saponins in the three organs was significantly different (P < 0.05). Overall, the average saponins content in leaves was 20.57 mg·g^-1^, which was two times the content in the roots (10.19 mg·g^-1^) and three times the content in the shoots (6.20 mg·g^-1^). The maximum saponins content was 24.45 mg·g^-1^ in leaves, >13.57 mg·g^-1^ in roots, and > 6.71 mg·g^-1^ in shoots.

**Fig 3:**
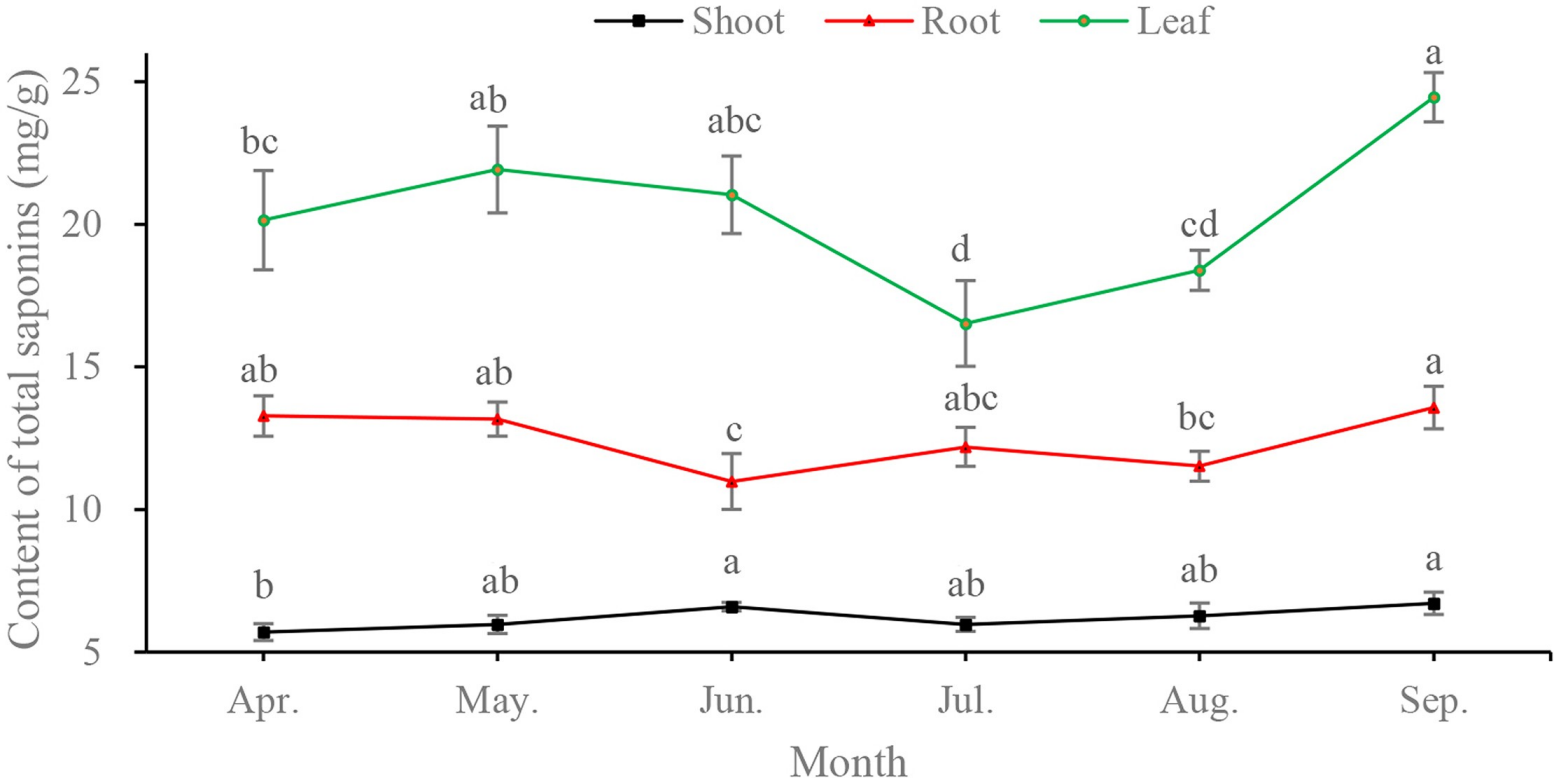
Dynamic patterns of saponins content in vegetative organs of *C*. *paliurus* at different months. Letters indicate statistical results at each month in shoot, root and leaf. The same letters indicate no significance according to Bonferroni test (P < 0.05).

Dynamic trends of total saponins during the growing months were observed in all three vegetative organs (Fig 3). The content of saponins fluctuated greatly in the leaf, varying from 16.52 mg·g^-1^ in July to 24.45 mg·g^-1^ in September. Saponins values in the shoot were the lowest and fluctuated less than both the root and leaf. The greatest values in all organs occurred in September before dormancy, and the minimum values occurred during the rapid growth period (from June to July). Interestingly, the second peak in all samples appeared during the initial growth in May.

## Discussion

### Histochemical localization of saponins in vegetative organs

Histochemical localization of saponins has been reported in perennial medicinal plants, such as *Panax ginseng*, *Gynostemma pentaphyllum*, *Bupleurum chinense*, and *Polygala tenuifolia* [17,19,20,27]. The study showed that distribution and accumulation of saponins in *C*. *paliurus* had tissue specificity and saponins accumulated mainly in the parenchyma of vegetative organs of *C*. *paliurus*, which was consistent with the reports provided by Liu et al. on *Gynostemma pentaphyllum* [27] and Teng et al. on *Polygala tenuifolia* [20]. We also found that the distribution of saponins was almost in the peripheral structure of vegetative organs, suggesting that saponins could be responsible for protecting plants against some pathogens and insects [29]. The localization of saponins in shoot showed that the transport of saponins in different organs could be accomplished by phloem. The high content of saponins in the main veins of leaves indicates that the main veins of leaves should be crushed during the processing of products in order to facilitate the release of active ingredients.

### Cytochemical localization of saponins in leaves

Saponins reacted with lead acetate and produced black complexes under electron microscope [23]. Based on the histochemical results in our study, the leaf of *C*. *paliurus* is an efficient material for the production of saponins. Thus, we conducted cytochemical localization on the leaf only. Localization of secondary metabolites at the cellular level has been the primary focus of studies that assess biosynthesis and physiological functions [30]. Our results of cytochemical localization in leaf of *C*. *paliurus* indicated the synthesis site of saponins in the cells and routes of saponins transfer in adjacent cells. The content of saponins and starch grains located in chloroplast increased gradually with the maturation of leaf. We speculate that saponins are mainly synthesized from photosynthate in chloroplasts, and then transport via plasma membrane system like endoplasmic reticulum vesicles. Chloroplasts, as a site of saponins synthesis and accumulation, has been reported in *P*. *ginseng* by Yokota [31], who found that Rb1 (a ginsenoside) was localized to the chloroplasts, peroxisomes and cytoplasm of leaf parenchymal cells. Furthermore, Yao et al. presented that terpenoid synthetase activity was remarkably increased on chloroplast membrane in the needles of *Pinus massoniana* seedlings when treated with MeJA [32]. Comparing the distribution of saponins in tender and mature leaves, the accumulation and synthesis of saponins are related to the structure and function development of chloroplast. Therefore, the photosynthetic efficiency should be considered to increase the accumulation of saponins and yield during the directional cultivation of *C*. *paliurus*. For black granules in the nucleus (Fig 2A and 2E), we are not sure whether they are saponins or other substances, and no publication declared the presence of saponins in nucleus. Further verification is needed to support this phenomenon.

### Dynamics of total saponins content in organs during the growing months

Determination of harvesting time for medicinal plants depends on the contents and yield of active ingredients in organs at different developmental stages [20,33]. As dynamic patterns presented (Fig 3), significant variations in the content of saponins among organs and growing months were found. Results showed that the maximum accumulation occurred in September, which was consistent with our findings from the histochemical localization. Usually, most of photosynthesis products are used as the source of primary growth (vegetative growth), leading to less metabolites accumulation in earlier growing stage. Interestingly, different from other plants [17,20], the content of saponins in the *C*. *paliurus* leaves maintained at a relatively high level, and kept increasing during the blossom period (late of April to May). The expression patterns of key genes related to the synthesis of saponins in yearling seedlings (data unpublished) supported this phenomenon to some extent, but more validation needs to be done on six-year-old plants. The increase of saponins content in earlier growing stage is supposed to be a stress reaction, owing to its sweet taste attracting more predators, and more secondary metabolites can play a role as chemical defense compounds against herbivores or microorganisms [30]. Subsequently, attributing to consumption of more photosynthate in developing fruits, a downward trend of saponins content in leaf occurred in June and July. With the leaf maturation and aging, an upward trend of saponins content appeared in August and September, suggesting that more photosynthetic products conversed into metabolites before dormancy. These results were consistent with the findings in *B*. *chinense* [17]. The high content of saponins in initial growth stage also provides guidance for leaf harvesting. Proper harvest in this period could prolong the time of *Cyclocarya paliurus* tea market and increase economic benefits.

Compared with roots and shoots, higher content of saponins in leaves indicated that leaves would be the main organ of saponins synthesis and storage. Consistent few content in shoot suggested that shoot might be the channel for saponins transportation.

High yield of saponins depends not only on the contents, but also on the biomass of the tree. As we know, for perennial deciduous woody plants as *C*. *paliurus*, more biomass of leaves is prone to be attained than shoots and roots. In common, the biomass of leaf in *C*. *paliurus* is increasing along with season from April to September. Owing to the largest leaf area and the lowest moisture content before leaf aging, the leaf biomass reaches the maximum in September. Moreover, leaf harvesting in September has little effect on tree growth in the following year. Comprehensively, we recommend that harvesting leaf in September is the efficient option of medicinal use for *C*. *paliurus*.

## Supporting information

S1 File Raw data for content of saponins. The data used for plotting after analysis is shown in the file.
(XLSX)
